# Elevated Salivary Levels of Oxytocin Persist More than 7 h after Intranasal Administration

**DOI:** 10.3389/fnins.2012.00174

**Published:** 2012-12-07

**Authors:** Marinus H. van IJzendoorn, Ritu Bhandari, Rixt van der Veen, Karen M. Grewen, Marian J. Bakermans-Kranenburg

**Affiliations:** ^1^Centre for Child and Family Studies, Leiden UniversityLeiden, Netherlands; ^2^Leiden Institute for Brain and Cognition, Leiden UniversityLeiden, Netherlands; ^3^Department of Psychiatry, University of North CarolinaCharlotte, NC, USA

**Keywords:** oxytocin, intranasal administration, saliva, 16 IU, behavioral effects, healthy female volunteers, daily oxytocin curve, ethics

## Abstract

We addressed the question how long salivary oxytocin levels remain elevated after intranasal administration, and whether it makes a difference when 16 or 24 IU of oxytocin administration is used. Oxytocin levels were measured in saliva samples collected from 46 female participants right before intranasal administration (at 9:30 a.m.) of 16 IU (*n* = 18) or 24 IU (*n* = 10) of oxytocin, or a placebo (*n* = 18), and each hour after administration, for 7 h in total. Oxytocin levels did not differ among conditions before use of the nasal spray. Salivary oxytocin levels in the placebo group showed high stability across the day. After oxytocin administration oxytocin levels markedly increased, they peaked around 1 h after administration, and were still significantly elevated 7 h after administration. The amount of oxytocin (16 or 24 IU) did not make a difference for oxytocin levels. The increase of oxytocin levels for at least 7 h shows how effective intranasal administration of oxytocin is. Our findings may raise ethical questions about potentially persisting behavioral effects after participants have left the lab setting. More research into the long-term neurological and behavioral effects of sniffs of oxytocin is urgently needed.

## Introduction

Intranasal oxytocin (OT) administration is used in numerous experimental studies to investigate the role of this neuropeptide in neural activation, information processing, and behavior (e.g., Insel, 1992; Carter, 2003; Parker et al., 2005; Feldman et al., 2007; Campbell, 2008; De Dreu et al., 2010; Naber et al., 2010; for reviews see Heinrichs et al., 2009; MacDonald and MacDonald, 2010; Bartz et al., 2011; Galbally et al., 2011; for a meta-analysis see Van IJzendoorn and Bakermans-Kranenburg, 2012). However, surprisingly little is known about the long-term effects of intranasal OT administration, and what minimal dose of OT might still be effective. Although 24 IU of OT seem to be standard in psychopharmacological experiments (with a range of 16–48 IU, see MacDonald et al., 2011; Van IJzendoorn and Bakermans-Kranenburg, 2012) there is no empirical evidence suggesting that lower doses would be less effective. Here we examine the influence of 16 or 24 IU OT on salivary OT levels in the course of the day in a sample of healthy female participants.

In the first study on levels of salivary OT after intranasal administration, Huffmeijer et al. (2012) found that OT levels remained highly elevated for more than 2 h. The study included 57 female participants who provided saliva samples right before, approximately $1\frac{1}{4}\text{h}$, and approximately $2\frac{1}{4}\text{h}$ after intranasal administration of 16 IU of OT or a placebo, in a double-blind, within-subjects design. Average levels of OT did not differ between conditions at baseline, markedly increased after 16 IU OT administration, and were still strongly elevated after $2\frac{1}{4}\text{h}$. Using a double-blind placebo-control within-subject design, Weisman et al. (2012) administered 24 IU OT or placebo 1 week apart to ten individuals (five females). They found strongly increased salivary OT levels 15 min after intranasal OT administration and OT levels remained high across the whole 4 h period that assessments took place.

The studies conducted thus far did not examine the effects of intranasal administration of OT after the end of a regular experimental session. In both studies levels of salivary OT were still highly elevated at the end of data-collection, after more than 2 h with 16 IU (Huffmeijer et al., 2012) and after 4 h with 24 IU (Weisman et al., 2012). Here we examine whether 16 and 24 IU doses of OT lead to similarly elevated levels of salivary OT, and we assess whether OT levels go down to baseline within 7 h after administration.

## Materials and Methods

### Participants

A total of 46 healthy female undergraduate students, aged 18–27 years (*M* = 19.77, SD = 1.53), without children of their own, took part in the study. Exclusion criteria were pregnancy, and use of steroidal or any other interfering medications. All participants were non-smokers and had not used any recreational drugs for at least 6 months before the experiment. Participants reported not having any current or past neurological or psychological disorders. The vast majority (90%) of the participants reported that they were in the luteal phase of their menstrual cycle. Their use of oral contraceptives was recorded in order to control for its influence on OT levels. The study was approved by the ethics committee of the Leiden University Medical Center.

### Design and procedure

Participants were asked to come to the laboratory for participation in an experimental session lasting about 2 h (to be reported elsewhere), and to stay around for 7 h in order to provide hourly saliva samples. The experimental task included a paradigm with happy and sad baby faces to be rated for, e.g., attractiveness (see Parsons et al., 2011). To minimize influences of diurnal variations in OT levels, all sessions started at 09:00 a.m. and ended between 16:30 and 17:00 p.m. with the collection of the 8th saliva sample. Participants were instructed to abstain from alcohol and excessive physical activity during the 24 h before the start of the session, and from caffeine on the day the session took place. Informed consent was obtained at the beginning of the session. Participants were not informed about the potential effects of OT under investigation, only about the possible side-effects they might experience (as was required by the ethics committee).

At the start of the session, a saliva sample was collected to assess baseline OT level, and participants completed a questionnaire. The participants then were asked to self-administer nasal spray containing either 16 IU (*n* = 18) or 24 IU (*n* = 10) of OT, or saline as placebo (*n* = 18) in a double-blind design in the presence of the experimenter. Thereafter, the participants provided another saliva sample every hour until a total of eight samples was reached. Participants were unable to discern whether they had taken active drug or placebo when asked for at the end of the testing session.

#### Oxytocin nasal spray

Oxytocin (Syntocinon^®^, OPG Groothandel, Oss, The Netherlands) was self-administered by spraying three puffs per nostril, each puff containing 0.067 ml (2.7 IU) for the 16 IU condition and 0.1 ml (4 IU) for the 24 IU condition The concentration of the oxytocin nasal spray was the same for 16 and 24 IU, with higher volumes used to deliver the higher dose.

#### Salivary OT

For each sample at least 1 ml of unstimulated saliva was collected into cryotubes using the passive drool method. Samples were immediately frozen and were stored at −20°C until batch assay. Level of OT in saliva was assayed using a commercially available kit as per the method previously described (Holt-Lunstad et al., 2008; Grewen et al., 2010). Prior to the enzyme immunoassay procedure, in keeping with the manufacturer’s strong recommendation, an extraction step was performed based on instructions accompanying the EIA kit available in February 2011 (ADI-900-153, Enzo Life Science, Plymouth Meeting, PA, USA). The result of this extraction was to concentrate the sample 3.2 times, increase precision, and reduce matrix interference. OT extraction efficiency was 94%, which was determined by spiking with a known amount of hormone and extracting this known amount along with the other samples. OT levels in extracted saliva were then quantified using the OT EIA, in which the endogenous OT hormone competes with added OT linked to alkaline phosphatase for OT antibody binding sites. After overnight incubation at 4°C, the excess reagents were washed away and the bound OT phosphatase was incubated with substrate. After 1 h this enzyme reaction, which generates a yellow color, was stopped and the optical density (OD) was read on a Sunrise plate reader (Tecan, Research Triangle Park, NC, USA). The intensity of the color at 405 nm is inversely proportional to the concentration of OT. The hormone content (in pg/ml) was determined by plotting the intensity of OD of each sample against a standard curve. Following correction for extraction, the lower limit of sensitivity was 1.0 pg/ml. Less than 3% of the samples fell below the lower level of sensitivity. These values were subsequently replaced with the lowest detectable level of 1.0 pg/ml. The intra- and inter-assay coefficients of variation were 7.56 and 8.20% respectively. The manufacturer reports that cross-reactivity with similar mammalian neuropeptides is less than 1%.

Missing data of individuals in the OT conditions were estimated using curve fitting of OT levels across the remaining time points using a logarithmic function. Missing data of subjects in the placebo condition were estimated with curve fitting on the basis of a linear function. Two different functions were used since they showed optimal fit in the two respective conditions (OT and placebo). Only two participants showed two missing data points; nine participants missed one data point.

### Statistical analyses

Statistical analyses were performed using SPSS statistics 19 software. Pearson correlations were computed between OT levels across all time points in order to examine stability of the salivary assessments. To test whether OT levels in saliva increased after intranasal OT administration, a repeated measures GLM analysis was performed with condition (placebo vs. 16 IU OT vs. 24 IU OT) as between-subjects factor, and time (baseline to 7 h after administration) as within-subjects factor. To control for a potential influence of the use of oral contraceptives (used vs. not used) on OT levels, this variable was included as additional (between-subjects) factor in a second GLM analysis. Greenhouse–Geisser corrections were performed when necessary.

## Results

Mean OT levels and correlations among OT levels across time points are presented in Table 1. Stability of OT in the placebo group was considerable; baseline values correlated 0.32–0.76 with OT levels later in the day. Stability in the OT group (16 and 24 IU combined) was more limited to adjacent time points (see Table 1).

**Table 1:** **Salivary oxytocin levels (M, SE) up to 7 h after intranasal administration and stability of OT levels over the day**.

Time	Placebo *n* = 18 *M* (SE)	16 IU *n* = 18 *M* (SE)	24 IU *n* = 10 *M* (SE)	Correlations (placebo under diagonal, combined OT groups above diagonal)							
				Baseline	1 h	2 h	3 h	4 h	5 h	6 h	7 h
Baseline	2.77 (0.65)	2.44 (0.58)	2.60 (0.82)	–	0.19	−0.04	−0.16	−0.21	−0.21	−0.22	0.12
1 h	3.88 (0.91)	446.56 (105.26)	157.36 (49.76)	0.32	–	**0.77**	**0.56**	0.24	−0.15	0.03	0.28
2 h	4.16 (0.98)	225.50 (53.15)	136.89 (43.29)	**0.76**	**0.55**	–	**0.56**	**0.38**	−0.02	0.06	0.24
3 h	3.27 (0.77)	100.40 (23.66)	57.71 (18.25)	**0.64**	0.37	**0.79**	–	**0.59**	−0.06	0.10	0.30
4 h	3.34 (0.79)	35.98 (8.48)	72.06 (22.79)	**0.68**	**0.52**	**0.84**	**0.77**	–	0.21	0.32	**0.68**
5 h	2.77 (0.65)	43.85 (10.34)	53.75 (17.00)	**0.65**	0.35	**0.87**	**0.89**	**0.82**	–	**0.51**	0.28
6 h	2.39 (0.56)	34.02 (8.02)	26.28 (8.31)	**0.56**	0.20	**0.74**	**0.63**	**0.88**	**0.75**	–	**0.38**
7 h	2.56 (0.60)	15.48 (3.65)	23.55 (7.45)	0.42	0.45	**0.63**	**0.71**	**0.67**	**0.69**	**0.56**	–

*Significant correlations (*p* < 0.05) in **bold***.

**Figure 1:**
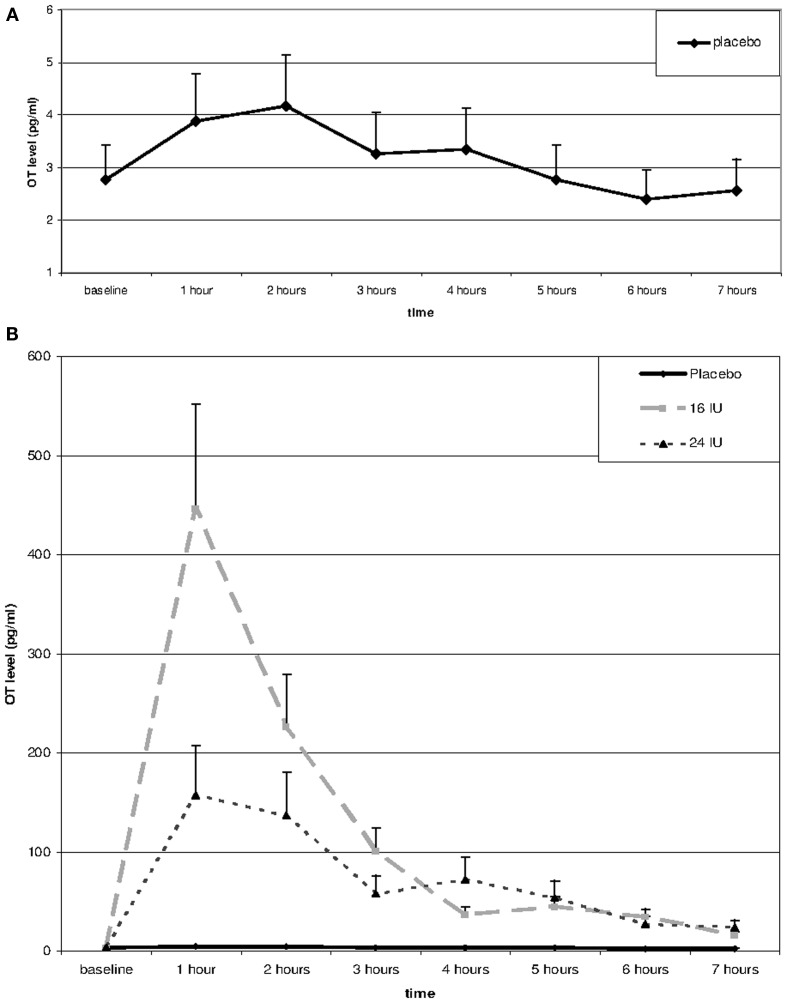
**Salivary oxytocin levels over the day in placebo (A,B) and after Administration of 16 or 24 IU oxytocin (B)**. Oxytocin or placebo was administered through nasal spray directly after the baseline assessment; subsequent assessments every hour after administration of oxytocin or placebo.

Repeated measures analysis of OT levels in the placebo group showed a significant effect of time, *F*(3.66, 62.14) = 7.14, *p* < 0.01, η^2^ = 0.30. A significant increase between baseline and 1 h later (*p* = 0.045) was followed by significant decreases between 2 and 3 h after baseline (*p* = 0.026) and between 4 and 5 h after baseline (*p* = 0.022), see Figure 1A.

Including the two OT conditions in the analysis, we found a significant interaction between time and condition, *F*(2.54, 54.49) = 5.20, *p* = 0.005, η^2^ = 0.20. Baseline OT levels were similar for the three groups, *F*(2, 45) = 0.28, *p* = 0.754, but at all time points after administration of either placebo or oxytocin (16 or 24 IU) participants in the oxytocin conditions showed much higher levels of salivary OT, see Figure 1B. Increases in mean OT levels were tenfold to hundredfold compared to the placebo condition, see Table 1. The effect had not faded out 7 h after administration, and at this point in time the difference was still significant, *F*(2, 45) = 7.15, *p* = 0.002.

The effects of 16 and 24 IU oxytocin were similar. There was no significant interaction between time and condition (16 IU vs. 24 IU), *F*(1.27, 32.95) = 2.06, *p* = 0.16, η^2^ = 0.07, and none of the time points showed a significant difference in OT level between participants in the 16 IU condition and participants in the 24 IU condition. At 1 h after administration the difference was maximal (*p* = 0.08), but the higher OT levels were found in the 16 IU group.

Including use of oral contraceptives in the analyses did not change any of the results.

## Discussion

Levels of salivary OT increased markedly after intranasal OT administration and remained elevated up to 7 h after administration, whereas in the placebo condition salivary OT remained at a consistently low level. Seven hours after administration the level of OT had not yet returned to baseline; in fact it was still six to tenfold higher than OT levels observed in the placebo condition. The lower dose of 16 IU OT did not show weaker effects over the day, if anything it tended to elevate the initial salivary OT levels more than the 24 IU dose.

In the placebo condition salivary OT levels were highly correlated over time, indicating an impressive individual stability of OT levels over the day. The increase in salivary OT between 1 and 2 h after placebo administration may be related to the experimental tasks, which included exposure to pictures of happy and sad baby faces (Parsons et al., 2011).

Elevated levels of salivary OT up to 7 h after administration are unlikely to support the idea that this would only result from movement of mucus from the nasal cavities back into the mouth, rather than reflect changes of OT levels in the brain that in several well-controlled oxytocin experiments using fMRI (Baumgartner et al., 2008; Riem et al., 2011, 2012) seem for example to down-regulate amygdala activation. Normally, a liter of fluid is generated daily, carried from the nose to the back of the throat and swallowed. Some of the mucus moves from the throat into the mouth and mixes with saliva. Part of the increase in salivary OT following nasal administration probably results from this direct movement of mucus but it is unlikely that this would account for the increased OT levels after 7 h. A mechanism parallel to sublingual or transdermal administration of various medicines and other exogenous substances seems more plausible (as suggested by Huffmeijer et al., 2012).

It remains to be shown how exactly intranasally administered OT enters into the brain or starts to influence brain neural activity along other pathways, and whether the effects are visible during at least 7 h not only in saliva but also in pertinent brain areas. The feed forward mechanism of the oxytonergic system, leading to more production of oxytocin with increased OT levels, may play an important role in the explanation of the persistently high OT levels. In natural circumstances, human lactation has been suggested to be subject to such a feed forward mechanism (Weisman et al., 2012), comparable to the Ferguson reflex, in which OT induced stimulation of the uterus facilitates the subsequent release of OT (Churchland and Winkielman, 2012). More warm physical contact has been shown to lead to higher OT levels in a linear fashion (Feldman et al., 2012), and a similar feed forward spiral of oxytocin levels may follow mutually reinforcing cooperation among individuals belonging to the same in-group (De Dreu, 2012). In rats, multiple oxytocin treatments have been shown to be more effective after each OT administration (Moberg, 2003). The feed forward mechanism of the oxytonergic system may lead to a self-perpetuating, elevated OT level after a first boost of intranasal OT, and in that way treatment with exogenous oxytocin may stimulate the “feed forward” release of the endogenous peptide. Nevertheless, basic research on the neurobiological pathway of intranasal oxytocin to pertinent parts of the brain is badly needed (Churchland and Winkielman, 2012).

The lack of a significant difference in salivary OT level between the 16 and 24 IU doses seems to indicate that the same concentration of the peptide was absorbed. The reason for slightly higher initial amounts of salivary OT in the 16 IU dose condition might be that the 16 IU dose saturates nasopharyngeal absorption resulting in maximal feed forward signaling, whereas higher doses might cause sub-maximal feed forward signaling. Spray volume may also have played a role, since 16 and 24 IU doses were administered as different volumes. A larger volume may result in more swallowing of the drug and thus lower availability for absorption (and less feed forward signaling). It should be noted that in a recent study Cardoso et al. (2012) found stronger effects of 24 IU of intranasal oxytocin on the cortisol response to vigorous exercise compared to a larger dose of 48 IU. For the dosage of oxytocin sniffs it is quite possible that “less is more,” but as noted before (e.g., Churchland and Winkielman, 2012) more research is needed to address the issue of optimal amounts of OT and the scheduling of the sniffs over time.

We assessed salivary OT at seven time points during the day with 1 h intervals. Because OT levels had not returned to baseline levels after 7 h, in future research assessments should cover a longer period of time, preferably including the night (as has been done for the diurnal curve of plasma OT levels, Forsling et al., 1998). The diurnal pattern of OT levels in plasma shows a peak around 2:00 a.m. with a sharp decrease during the second half of the night and an almost flat pattern during the day. Future studies should examine this pattern with salivary OT levels in placebo as well as in OT administration conditions in order to know at what point in time the effects of intranasal administration of OT fade out and when OT levels get back into the regular diurnal pattern.

Increased levels of oxytocin until at least 7 h after the administration present possibilities for designing experiments on the impact of oxytocin administration on cognitive functions, emotions, and social behavior over longer periods of time. More empirical evidence is needed to determine whether the increased salivary OT levels also translate to measurable neurophysiological changes over time. Intranasal oxytocin administration is increasingly used in clinical trials on a variety of psychiatric disorders (Meyer-Lindenberg et al., 2011) and in some cases the long-term effects of a single dose seem promising. At the same time studies on the long-term consequences of oxytocin administration may also clarify emerging ethical questions involved in the use of oxytocin in brief experiments. Although MacDonald et al. (2011) showed the absence of detrimental side-effects of various amounts of oxytocin administered in a large number of experiments on clinical and non-clinical samples, it remains to be seen whether the positive behavioral effects of oxytocin on donating and other forms of (parochial) altruism persist across a longer period of time and affect interactions in the natural setting outside the laboratory. For some subjects these “positive” effects might be unwanted and they may have to be informed before giving consent.

In conclusion, we found that a single intranasal administration of OT induces elevated levels of salivary OT up to 7 h after OT administration, way beyond the period of a few hours in which neurobehavioral effects of OT are commonly observed in psychopharmacological studies. Because we do not know whether the prolonged elevation of OT levels leads to long-term changes in brain activation, cognition, and behavior, our findings raise ethical questions about the use of intranasally administered OT without provisions for the aftercare of the participants involved in OT experiments.

## Conflict of Interest Statement

The authors declare that the research was conducted in the absence of any commercial or financial relationships that could be construed as a potential conflict of interest.
